# Total humerus replacement with reverse shoulder design for non-oncologic indication: A case report

**DOI:** 10.1016/j.ijscr.2023.108326

**Published:** 2023-05-24

**Authors:** Manh Nguyen Huu, Quyet Tran, Viet Vu Duc, Dung Tran Trung

**Affiliations:** Department of Orthopaedic Surgery, Vin University, Hanoi, Viet Nam; Orthopaedic and Sports Medicine Center, Vinmec Healthcare System, Viet Nam

**Keywords:** Total humerus megaprosthesis, Reconstruction of large skeletal defect, Osteomyelitis, cancer bone

## Abstract

**Introduction and importance:**

Reconstruction of large skeletal defects pose significant challenges for orthopedic surgeons, particularly in cases of chronic skeletal defects where the surrounding structures differ substantially from the original anatomical structures, further complicating management.

**Case presentation:**

A 54 year old male patient presented with a large skeletal defect after osteomyelitis surgery. The treatment of choice for this case was reconstruction using a total humerus megaprosthesis. The prosthesis was custom-designed with a reversed shoulder joint and a total elbow joint, which were 3D printed using CT-Scan imaging.

**Clinical discussion:**

A short-term follow-up revealed improvements for the patient in arm functionality and expectation-based satisfaction at 6 months post-surgery.

**Conclusion:**

Total humerus megaprosthesis joint replacement may be a promising option for treating chronic humeral defects.

## Highlights

• Large bone loss of humerus after osteomyelitis is quiet rare
• Total humerus replacement is not common procedure for non-oncologic indication
• a modified design of total humerus implant with reverse shoulder could give a better result for patient.

## 1 Introduction

Large skeletal defects of the arm can arise from different causes, including trauma, limb-sparing surgery associated with tumors or bone diseases such as osteomyelitis. To this day, reconstruction of large skeletal defects and limb rehabilitation has continued to pose significant challenges for orthopedic surgeons. Several surgical approaches have been developed, including bone grafting, distraction osteogenesis, Masquelet technology, and prosthesis implantation, each with their own set of advantages and disadvantages [1].

Autografts such as pelvic grafts and fibulas with vascular stalks are considered the gold standard for post-traumatic non-extensive bone defects due to their excellent healing rate, reduced risk of graft rejection, fewer complications, and overall better functionality compared to allografts [2]. For patients with bone cancer, reconstruction of the bone structure after limb-sparing surgery can be achieved through endoprosthesis, allograft, or allograft-prosthesis. However, none of these options are superior to others, as post-operative limb function remains limited, and the risk of complications such as infection, loose grip, structural failure, and local recurrence remains apparent [3].

Megaprosthesis has been used in cases of large skeletal defects since the 1970s and has gradually gained popularity up to the present day. N. Mayilvahanan et al. reported a case series of 63 patients with humeral head cancer who underwent extensive tumor resection and custom megaprosthesis joint replacement, resulting in functional satisfaction in 78 % of cases. However, complications such as artificial sub-dislocations and joint loosening were encountered [3]. In this case report, we present a patient with a large humeral defect who underwent reconstruction surgery using total humerus megaprosthesis after bone-resection surgery due to infection. This case report has been reported in line with the SCARE Criteria [4]. Ethical Approval was waived by the authors' institution.

## 2 Presentation of case

A 54-year-old male patient, who is self-employed, presented with a medical history of an injury sustained 40 years ago, when he fell and suffered a fracture of the right arm in a work-related accident. The patient subsequently underwent a surgical procedure for internal fixation of the fracture. However, he developed a post-surgery infection that necessitated two additional surgeries to manage the inflammation and remove a significant portion of the humerus bone.

Following the surgical interventions, the patient had to adjust to living with a large skeletal defect in his arm for over 40 years, which had a profound impact on his quality of life, leading to several limitations in his ability to control the movement and form of his arm, particularly in flexion and extension. As a result, the patient sought medical attention to address the issue and enhance the functionality of his arm in daily activities.

The preoperative assessment revealed that the right arm was retracted with a length difference of 17 cm compared to the contralateral arm. The original humeral head was found to be anteroinferiorly dislocated; however, the deltoid muscle strength remained good. The shoulder joint had limited abduction and flexion (up to 70°), relying on the musculoskeletal system's integrity around the shoulder joint, particularly the deltoid muscle. The arm could not be maintained in an elevated position due to the humerus loss. Elbow movement was mostly accompanied by shoulder movement, and the joints were partially stiff with no neurological deficit detected. Despite the shoulder dislocation and missing humerus, the patient has adapted to using his hands for basic activities, such as dressing and getting things off the shelf, despite the fact that these shoulder movements require more force from his torso.

Preoperative X-ray and CT images revealed the humeral head's dislocation and narrow elbow joint space. Additionally, the elbow MRI revealed synovial thickening. The length from the greater tuberosity to the lateral epicondylar was measured to be 12.83 cm while standing and 15.72 cm while holding weights. An MRI scan of the shoulder was assigned to evaluate the condition of the rotator cuff tendon and the deltoid muscle to provide the best solution for the patient. The rotator cuff muscle group was found to be attached to the remaining humeral head, but the tendon was considerably contracted, and the surrounding muscles had atrophied with fatty degeneration. The deltoid muscle, however, remained intact.

This is a challenging orthopedic case that requires careful consideration due to the complexity of the patient's condition. The patient has been able to compensate for his limited shoulder and elbow mobility by relying on passive arm swing during daily activities. However, surgery has been proposed as a means of improving the patient's voluntary shoulder and elbow mobility.

**Fig. 1:**
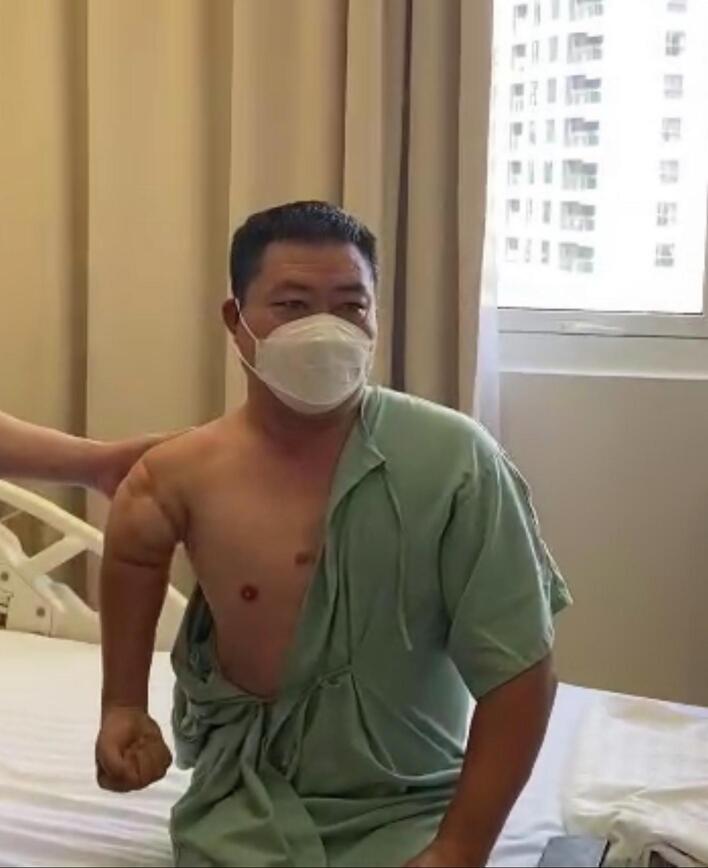
Preoperative image of a chronic large skeletal defect in the right arm with a major retraction of soft tissue (figure a), arm abduction relies on deltoid muscle (figure b).

**Fig. 2:**
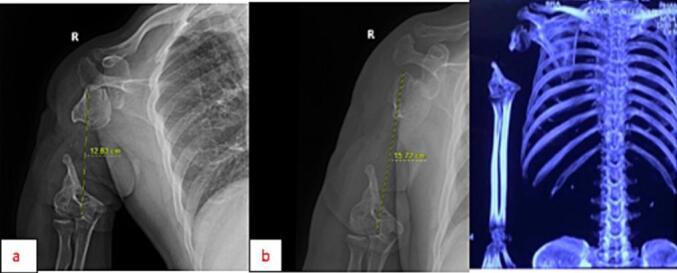
X-ray of the patient's right shoulder not bearing weights (figure a), bearing weights (figure b) and CT-scan of his right arm.

**Fig. 3:**
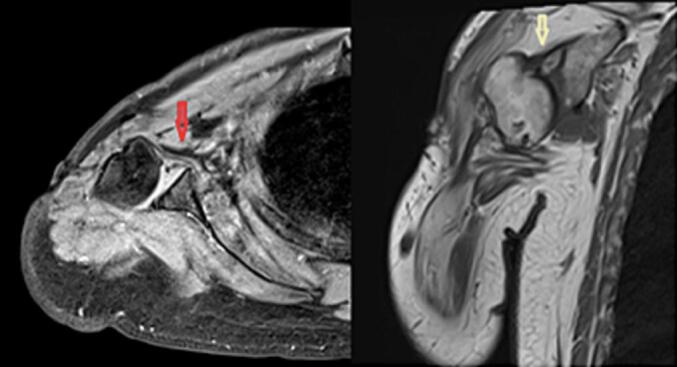
MRI image of the patient's shoulder joint, subscapularis tendon (red arrow), supraspinatus tendon (gray white arrow).

**Fig. 4:**
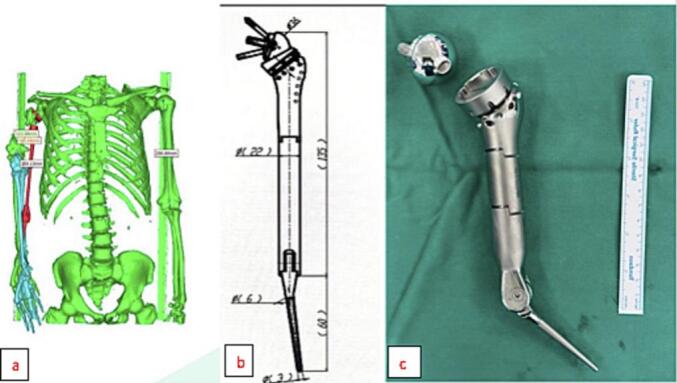
3D rendering to calculate joint dimensions (figure a). Modular megaprothesis joint design (figure b) Joints are manufactured from the customized design (figure c).

**Fig. 5:**
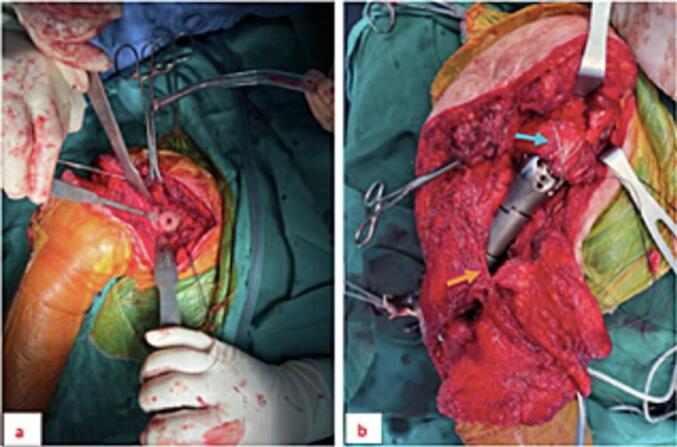
Preparation of the glenoid fossa. Important muscle tendons, such as the pectoralis major tendon, are sutured and marked with sutures (figure a). After the placement of the artificial joint (figure b), the radial nerve is peeled off. Exposed separation (orange arrow), the rotator cuff attachment site on the large tubercle, and the minor tubercle of the humerus are preserved and sutured to the proximal joint (blue arrow).

**Fig. 6:**
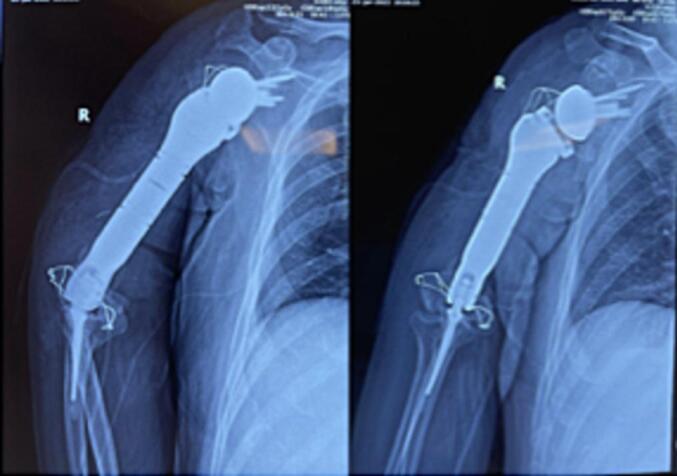
Postoperative X-ray of the patient.

**Fig. 7:**
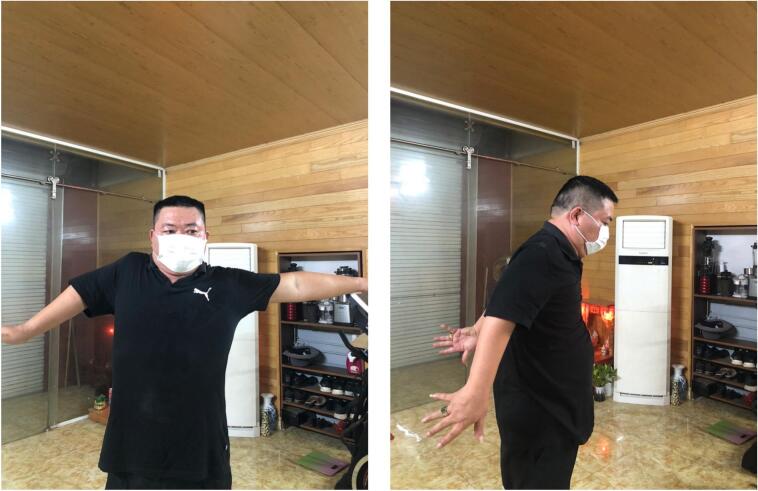
Significant improvement in shoulder joint movement at the 6-month post-operative follow-up.

Due to chronic shoulder dislocation and elbow stiffness, the remaining ends of the humerus are insufficiently short, with the upper part consisting only of the humerus head, and the lower part measuring only 2 cm in length compared to the epicondyle. Bone grafting was not considered due to its inability to address the chronic shoulder dislocation and elbow stiffness. MRI imaging showed severe atrophy and fatty degeneration of the rotator cuff muscle group, which makes solely relying on the full shoulder joint risky, as the possibility of rotator cuff tendon function deficiency would be considerable. Therefore, a reverse shoulder joint replacement was suggested as the preferred option. The patient's elbow joint function has been lost for a long time, leading to partial stiffness and a clearly narrow joint space. Therefore, a full elbow joint replacement was suggested with the addition of a 4 cm module in the middle of the two replacement joints to fully optimize the arm length for the patient. 3D rendering software was used to calculate and measure the design, ensuring that the joint length is within the arm's length range without weights (Fig. 2), and the joint is properly fitted without being too tight or too loose. Due to the manufacturer's module length limitation, a shoulder joint arm length of 135 cm and an elbow joint stem length of 60 cm were selected as the most feasible options. (See Fig. 1, Fig. 3, Fig. 4, Fig. 5, Fig. 6, Fig. 7.)

Surgical technique: The patient was placed under endotracheal anesthesia and positioned in a 45-degree Beach chair. A surgical incision was made from above the coracoid process along the deltoid groove down the back of the arm past the elbow. Due to the complex interlacing of soft tissue, nerves, and blood vessels resulting from previous operations, the surgical path no longer followed the anatomical landmarks. Dissection was performed carefully to avoid damaging important structures. The surgical procedure was divided into two main stages: shoulder replacement and elbow replacement.

During the first stage of shoulder replacement, we retained the attachment point of the rotator cuff tendons to the humeral head, and were precise in determining the deltoid and pectoralis major attachment points. We minimized separation of the attachment sites of these muscles from the surrounding muscle, ensuring their attachment to the surrounding muscle fascia. After the artificial joint was replaced, the attachment of the rotator cuff to the bone was sutured into the holes designed above the reversed total shoulder joint.

In the second stage of elbow joint replacement, we followed standard protocol and retained the two inner and outer condyles, fixed them with steel thread, and bridged the bone graft between the two brachial condyles. Maximizing bone salvage in the elbow area facilitated a more stable elbow joint with better load-bearing capacity and durability. After placing the reversed shoulder joint and elbow joint, a module was placed in the middle to prevent the radial nerve from being overstretched.

After surgery, the patient was followed up in the ward; the motor and sensory functions of the right wrist were good, and the sensor functions of the right arm were normal. The patient was stable and, therefore, discharged after 5 days.

The postoperative rehabilitation program involved static muscle contraction, flexion, extension, pronation, and supination of the forearm, along with machine-assisted shoulder exercises within a 90-degree range for the first six weeks, along with cryotherapy. Over the following six weeks, the patient gradually transitioned from passive to partially active exercises. From the third month onward, the patient engaged in fully active muscle exercises, with increasing resistance over time. Deltoid muscle exercises were emphasized for their importance in shoulder function.

The patient were scheduled to be re-evaluated at these post-operative follow-ups: 2 weeks, 4 weeks, 6 weeks, 3 months, 6 months, and 12 months.

## 3 Current post-operative six-month follow-up findings

Right shoulder range of motion: flexion of 110°, extension of 40° and abduction of 90°,

Right elbow range of motion: flexion of 120°, extension of 0°, and good right forearm pronation.

On the post-operative X-ray at 6 months:

The patient's function improved greatly in terms of active movements of the shoulder and elbow in daily activities. The patient was overall very satisfied with the result of the surgery.

## 4 Discussion

The primary causes of large skeletal defects are trauma, bone tumors, and bone infections. The goal of surgical interventions in these cases is to retain the limb, avoid amputation, and restore maximum limb function. However, the optimal surgical approach is still a topic of debate. Autograft and allograft are known to offer long-term advantages, such as bone tissue regeneration and improved function and kinetics. Nonetheless, they can be associated with prolonged surgical time and complications such as fractures, infections, non-healing bone, and osteoarthritis. Pelvic bone autograft can promote bone healing and is cost-effective, but its use is limited to small skeletal defects due to the restricted bone volume. In order to solve the problem of large skeletal defects, one method has been proposed: using vascularized and nonvascularized pedicled fibular grafts. Taylor et al. have reported and successfully implemented this method since 1975 [5] The fibular graft method retains many of the benefits of autograft but has several drawbacks, including size matching with the bone defects, prolonged surgery time, and an additional microsurgery to harvest the fibula with a vascular pedicle.

In a comparison of vascularized and non-vascularized autologous fibular grafting, Schuh et al. observed no significant difference in function but higher bone healing-related surgical rates in the vascularized group [6]. In cases of diaphysis cancer, the removed bone can be treated with methods like pasteurization, autoclaving, gamma irradiation, or cryotherapy and then reassembled in the original position. Although this processed bone belongs to the patient, it can reduce bone healing rate. Allograft offers an alternative approach as it can be obtained from other donors (allogeneic) or animals (xenogeneic) and can provide immediate bone shape recovery. However, several limitations such as graft rejection, the risk of transmitting infectious diseases such as HIV, infection, and non-healing bone exist. Mankin et al. reported an infection rate of up to 12.8 % in a series of 945 patients with allogeneic bone grafts. Hornicek et al. also recorded a bone failure rate of 17.2 % in the allograft group, which increased among patients receiving chemotherapy [7].

In recent years, advancements in 3D bioprinting bone technology have shown promise as a potential solution to the problem of graft rejection. Hydroxyapatite, tricalcium phosphate, dicalcium phosphate, calcium sulfate, and bioactive glasses are commonly used materials for bone-graft substitutes, which exhibit high availability, biocompatibility, and strength similar to the original bone while promoting bone formation. The size and shape of the graft can be precisely calculated through software, creating a personalized product for each patient. However, the use of biological reconstruction grafts for large skeletal defects in the epiphysis can be challenging due to joint surface incompatibility and graft fixation issues.

Megaprothesis reconstruction is an alternative option for dealing with large skeletal defects, particularly in cases of epiphysis or extensive diaphysis cancer accompanied by joint injuries. This technique offers benefits such as personalized design, early mobility, fewer complications, and independence from bone healing rate, unlike biological reconstruction. However, long-term complications may arise, including aseptic loosening, dislocation, infection, and revision surgery in cases of children [8]. Nevertheless, revision surgery can effectively address these complications.

Megaprosthesis have been successfully used for reconstruction after extensive resection of osteosarcoma [9] and large skeletal defects [10] since the 1990s, and they continue to be developed. Advancements in science and technology have enabled the use of 3D printing to create artificial joint products of the appropriate size for patients. In the current clinical case, the patient's entire shoulder-arm structure was reconstructed using CT scans, and joint length, joint component size, suture position, rotator cuff tendon attachment point site, and deltoid muscle measurements were taken by 3D lab technicians. These parameters and CT scans were sent to the manufacturer to create the finished product. The critical point in the calculation process is choosing the appropriate arm length to accommodate the soft tissue tensile range while ensuring that it is neither too loose nor too tight. A probable solution for this problem is for the patient to hold heavy weights in order to determine his maximum arm length.

Anatomical endoprosthesis for upper epiphysis injuries of the arm was first introduced by Charles Neer II in the 1950s to the 1970s [11]. Despite numerous design improvements, outcomes have remained relatively limited in terms of shoulder joint function and range of motion [12,13], mainly due to rotator cuff functional loss and artificial joint instability. To address this problem, reverse shoulder prosthesis were developed to significantly improve shoulder function, range of motion, and patient satisfaction, while reducing common prosthetic joint complications such as loose joints and dislocations. Jung Youn Kim et al. tracked and assessed the clinical outcomes of 98 total reverse shoulder joints; the mean Constant score increased from 35.4 to 57.8; the UCLA score increased from 13.4 to 28.8, and scapular notching was the most common complication in 17 cases [14]. Although our patient's deltoid muscle function is intact, the rotator cuff muscle group is difficult to assess clinically. Magnetic resonance imaging shows a small and shrinking preoperative muscle group with a changed globular structure due to chronic shoulder dislocation. As a result, we have selected the reverse shoulder joint as the optimal approach.

In 1972, Dee developed and reported the method of total elbow replacement which is primarily indicated for severe rheumatoid arthritis, complicated elbow fractures, severe osteoarthritis, post-traumatic osteoarthritis, post-traumatic large skeletal defects, and reconstruction after surgery for primary bone cancer, elbow metastases, and hemophilic arthropathy [15]. Various artificial elbow joint designs have been created, including the unlinked design with separate ulnar and humerus implants, linked semiconstrained design featuring a central cylindrical bearing and two side bearings, semiconstrained condylar-bearing design with a hemispherical shape, and convertible design that allows surgeons to choose between nonconstrained and semiconstrained prostheses [16]. In cases of large skeletal defects after trauma or osteotomy, modular megaprosthesis, allograft, autograft, or allograft-prosthesis can be used for elbow joint reconstruction. However, dissatisfaction with the graft technique arises due to prolonged healing time, risk of non-healing, and periprosthetic infection. Rodolfo Capanna et al. reported a modular megaprosthesis reconstruction of the elbow joint in 36 patients (31 oncological and 5 non-oncological), showing good to great elbow function at 66.7 %; elbow range of motion greater than 100° reached 66.7 %; the survival rate of the implant was 93 %; 6 patients had complications, including radial palsy, ulnar palsy, infection, and disassembling of the articular prosthesis component [17]. The outcome in terms of elbow joint function is on par with other methods such as allograft-prostheses and osteoarticular allografts, but the complication rate is lower [18]. Tran Trung Dung and colleagues utilized 3D printing technology to develop personalized linked megaprosthesis joint in two cases of patients with post-traumatic large skeletal defects, where the 14-month follow-up revealed good functional performance: The Mayo Clinic score was 97.5, and the ROM was 135°-0°-0° [19].

In order to provide better elbow function for our patient with a stiff elbow joint and short remaining epicondylar bone, we opted for a linked semiconstrained total elbow replacement, as bone grafting was deemed too risky. It is important to note that total elbow joint replacement has several disadvantages, including aseptic loosening (12.9 %), infection (13.3 %), implant failure (0.65–1.2 %), and revision surgery (18 %) [20].

The primary goal of the surgery was to improve shoulder, arm, and elbow mobility, allowing the patient to keep his arm in an elevated position. While the surgery was initially successful, the limitations and potential complications of total elbow joint and reverse shoulder joint require ongoing monitoring.

## 5 Conclusion

The use of a modular megaprosthesis replacement for the entire humerus is a potential approach to address large skeletal defects close to the humeral epiphysis, assisting in functional rehabilitation of the elbow and shoulder joints. Nonetheless, the procedure is complex, necessitating a high degree of surgical expertise and pre-operative planning to select the most suitable joint replacement method.

## Provenance and peer review

Not commissioned, externally peer-reviewed.

## Consent

Written informed consent was obtained from the patient for publication of this case report and accompanying images. A copy of the written consent is available for review by the Editor-in-Chief of this journal on request.

## Ethical approval

Ethical approval is exempt/waived at our institution

## Funding

The authors declare that sponsors had no such involvement.

## Author contribution

- DTT contributed to perform the operation, revising, and approval for publishing.
- MNH, QT contributed to assist the operation, data collection, analysis and interpretation, manuscript drafting.
- VVD contributed to data collection, analysis and interpretation, manuscript drafting.

## Guarantor

Professor Dung Tran Trung MD, PhD.

## Research registration number

This is cases report so we do not need to register.

## Declaration of competing interest

The authors declare that there is no conflict of interests regarding the publication of this paper.

## Appendix A Supplementary data

Video 1 Movement of patient before surgery. Video 1

Video 2 Movement of patient before surgery Video 2
